# Is the Use of Surface-Enhanced Infrared Spectroscopy Justified in the Selection of Peptide Fragments That Play a Role in Substrate–Receptor Interactions? Adsorption of Amino Acids and Neurotransmitters on Colloidal Ag and Au Nanoparticles

**DOI:** 10.1021/acs.jpcb.1c00546

**Published:** 2021-03-01

**Authors:** E. Proniewicz, A. Ta̧ta, E. Iłowska, A. Prahl

**Affiliations:** † Faculty of Foundry Engineering, AGH University of Science and Technology, 30-059 Krakow, Poland; ‡ Faculty of Chemistry, University of Gdansk, Wita Stwosza 63, 80-308 Gdansk, Poland

## Abstract

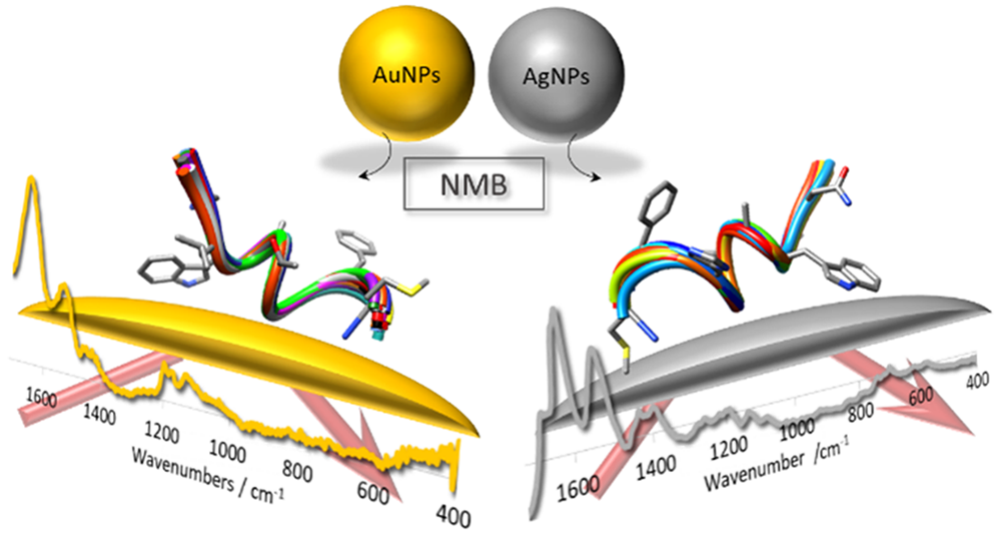

This paper describes an application of attenuated total reflection Fourier transform infrared spectroscopy (ATR-FTIR) and surface-enhanced infrared spectroscopy (SEIRA) to characterize the selective adsorption of four peptides present in body fluids such as neuromedin B (NMB), bombesin (BN), neurotensin (NT), and bradykinin (BK), which are known as markers for various human carcinomas. To perform a reliable analysis of the SERIA spectra of these peptides, curve fitting of these spectra in the spectral region above 1500 cm^–1^ and SEIRA measurements of sulfur-containing and aromatic amino acids were performed. On the basis of the analyses of the spectral profiles, specific conclusions were drawn regarding specific molecule–metal interactions and changes in the interaction during the substrate change from the surface of silver nanoparticles (AgNPs) to gold nanoparticles (AuNPs).

## Introduction

Vibrational spectroscopy (infrared absorption and Raman) is a widely used, reliable, and powerful method for studying conformational changes and molecular interactions and for unambiguously identifying and characterizing a variety of molecules by their vibrational fingerprint. However, in conventional form, it does not provide sufficient sensitivity for trace concentrations and thin molecular layers (usually a few pmol/cm^2^), since most (bio)organic molecules absorb radiation in the mid-infrared range (2.5–25 μm) relatively poorly and do not scatter electromagnetic radiation effectively. This leads to a limitation of the application range of vibrational spectroscopy based on the detection of chemical traces (food safety, detection of hazardous substances, or biosensors). To overcome these limitations, surface-enhanced techniques of this method have been developed and applied using highly concentrated fields in the vicinity of resonantly excited plasmonic structures. The surface-enhanced technique also overcomes another limitation of infrared spectroscopy, where the extremely high IR absorption of water prevents the direct use of an aqueous medium in IR measurements since the enrichment of the sample along the metal surface reduces the water content in the observed volume.

In the early 1980s, Hartstein, Kirtley, and Tsang first observed the phenomenon of surface-enhanced infrared absorption,^1^ which was named SEIRA in 1991 by analogy with surface-enhanced Raman spectroscopy (SERS) developed in 1974.^2^ To date, however, SEIRA has not gained the importance of SERS due to the lower signal enhancement (compared to SERS),^3^ which is typically 10^1^–10^3^ when the molecule is adsorbed on or near (10 Å or less) rough surfaces of a variety of metals. The first SEIRA studies used mainly noble metals (Ag,^4^ Au,^5^ and less frequently Cu^6^). Later reports showed the possibility of using other metals,^7−14^ semiconductors,^15^ and polar dielectric nanostructures.^16^

The SEIRA effect is mainly studied on chemically deposited and vapor-deposited metal island films, nanoparticle decorated films,^5^ periodic array-based substrates (consisting of particles and holes),^17^ and less frequently metal sols.^18^ Metal films consist of growing and converging isolated particles that eventually form a continuous film. During this process, the signal from the adsorbate is strongly enhanced until the percolation threshold is reached (or close to it), whereupon the signal strength decreases until it completely disappears once a continuous film is formed.^19^ Signal enhancement on these substrates is mainly based on the off-resonance mechanism. For resonance enhancement to take place in the infrared region, the structure must have the right size, which unfortunately is not possible for metallic island layers (an island size much smaller than the wavelength of the adsorbed light is necessary to produce amplification). The solution in this situation is to use other metal substrates, such as metal sols, which are also important for other reasons; e.g., they are relatively fast, easy, and inexpensive to obtain; they allow reproducibility of signal enhancement due to synthesis procedures that ensure low dispersion of the nanoparticle diameter in the sol and do not require strict topological control; they can be used in transmission mode without using complicated optical systems, and the sample is attached to a surface of colloidal nanoparticles before measurement. The latter is particularly important in the case of metals with photocatalytic properties, whose properties can be inhibited by functionalizing their surface with biological material, and in the context of the development of hybrid biodevices (biomolecules associated with the substrate that actuates them). This concept assumes the possibility of triggering and testing the properties of the adsorbate at the interface created between the biomaterial and the substrate. However, despite numerous studies on optical biosensing with SEIRA, there are still too many unknowns (e.g., related to controlled morphology and reproducibility) that preclude routine use of this technique in biology and medicine. For this reason, we have undertaken the current research to achieve improved absorption using commonly available and homogeneous, in terms of shape and diameter, Ag and Au colloids (due to the aforementioned advantages), which will allow the broader and routine application of SEIRA. At the same time, we extend relatively little knowledge about the use of SEIRA for the study of biological systems, the detailed properties of which we present. The choice of biological systems such as neuromedin B, bombesin, neurotensin, and bradykinin was dictated by the fact that they are the natural ligands of metabotropic seven-transmembrane G-protein-coupled receptors (GPCRs), which are overexpressed on the surface of many malignancies, making these receptors (when interacting with their ligands conjugated to metal nanoparticles) potentially available as receptor-positive cancer markers in early diagnosis for tissue lesions detection and anticancer therapy.^20,21^

The supplemented information in the databases on spectroscopy of amino acids and neurotransmitters will also allow a more accurate interpretation of the spectra of complex molecules, such as peptides and proteins. This is because many research groups have focused on the preparation of new substrates and the determination of signal enhancement and SEIRA mechanism using adsorbates,^22^ which usually contain carbonyl and thiol groups^23^ or adsorbates in the form of small and/or symmetric molecules^3,5,10,11,14,24−28^ and less frequently thin polymer films.^29−34^ Few literature reports indicate the use of SEIRA in the study of biosensors,^35−51^ in cancer drug research (cis-platinum and doxorubicin),^52^ and as a diagnostic criterion for cancer.^52,53^

SEIRA is concerned with those bands of the adsorbate whose vibrational modes contain a dipole component perpendicular to the surface.^54^ The enhancement of these bands depends on three main factors, which have been classified as “chemical” and “physical” based on their origin.^17^ Physical factors are associated with an enhancement of the electromagnetic field near a rough metal surface or metal island films and can be divided according to whether the frequency of the plasmon matches the frequencies of the adsorbate vibrations (on-resonance contribution) or whether the plasmon frequency is far away and only a small direct interaction between plasmons and vibrations occurs.^17^ In the infrared region, the frequency of the plasmon can be tuned by morphology (size, shape, particle density, and average thickness) of the metal islands/layers, the surface structure of the supporting substrate, the experimental conditions, and the surrounding medium.^3,55,56^ In the case of chemical factors, it is assumed that chemical bonding leads to changes in the electronic structure of the adsorbate and thus changes the dipole transition moment of the adsorbate vibrations, making vibrations not allowed in the infrared spectrum infrared active.

Other factors affecting infrared absorption include the chemical composition of the adsorbate; infrared absorption occurs mainly for vibrations of polar groups with large dipole moment gradients. Because of this phenomenon, this work also extends the knowledge of previously published works on SERS sensing.^57^

## Materials and Methods

### Adsorbates and Colloids

Unprotected amino acids were purchased from Sigma-Aldrich, Poland, and used without further purification (99,99% purity).

Neuromedin B (NMB), bombesin (BN), neurotensin (NT), and bradykinin (BK) were synthesized via the solid-phase method using the Fmoc strategy and starting from Fmoc-Wang resin (GL Biochem Shanghai, 1% DVB, 100–200 mesh) (see Supporting Information for details).

Gold and silver colloidal solutions (spherical nanoparticles with a diameter of 20 nm (Au) and 40 nm (Ag)) were purchased from Merck (Poland).

### ATR-FTIR and SEIRA Measurements

Before SEIRA measurements, each peptide (30 μL of an aqueous peptide solution) was immobilized on colloidal suspension (10 μL). The mixture peptide/colloidal nanoparticles were then deposited on a diamond ATR adapter and allowed to dry. Unbound peptide molecules were removed through washing with deionized water and allowed to dry. The process was repeated three times.

The spectra were recorded on an FTIR Thermo Scientific Nicolet 6700 spectrometer equipped with a diamond ATR accessory. Measurement conditions were the following: a resolution of 4 cm^–1^ and 128 scans. SEIRA spectra were recorded three times at three different locations on each substrate surface. The SEIRA spectra of a given adsorbate on a given substrate were almost identical except for small differences (up to 5%) in some band intensities. No spectral changes that could be associated with the decomposition of the sample were observed during these measurements.

### Spectral Analysis

Multiple bands not separated were fitted using a GRAMS/AI program (Galactic Industries Co., Salem, NH). Briefly, a 50/50% Lorentzian/Gaussian band shape for all bands was assumed and fixed. The number of bands, their initial wavenumbers, bandwidths (full width and half-maximum), and intensities were selected based on results from previously published IR studies and careful examination of spectra obtained in this work.

### Peptide Structures

3D peptide structures were obtained by GaussView 3.0 (Gaussian Inc., 2000–2003) and UCSF Chimera 1.8.1. (by Regents of the University of California, 2000–2013) software.

## Results and Discussion

### Sulfur-Containing Amino Acids

**Figure 1:**
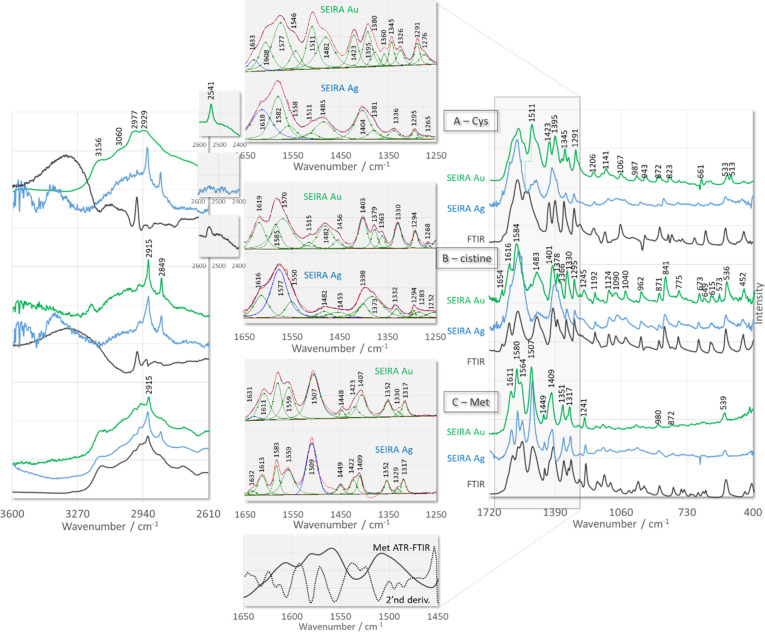
ATR-FTIR (black line traces) and SEIRA (with curve fitting results) spectra of sulfur-containing amino acids adsorbed onto the surface of AgNPs (blue line traces) and AuNPs (green line traces).

Figure 1 shows the SEIRA spectra of sulfur-containing amino acids (e.g., l-cysteine (Cys), cystine, and l-methionine (Met)) immobilized on the surface of AgNPs and AuNPs. ATR-FTIR spectra are also included in this figure to highlight the changes between SEIRA and ATR-FTIR spectra. The SEIRA spectra in the spectral region below 1000 cm^–1^ are not analyzed in detail; instead, the observed bands and their assignments are summarized in Table 1. The ATR-FTIR spectra are also not discussed here as they are consistent with IR spectra published in the literature.^58−63^ However, there are some differences in the intensity and position of the bands. The ATR-FTIR spectra have much stronger bands at lower wavelengths than at higher wavelengths compared to the transmission FTIR spectra. This is because the penetration depth (apart from the refractive indexes of the sample and ATR crystal and the radiation incidence angle) depends on the radiation wavelength and increases with increasing wavelength. The wavenumber shift results from the amount of reflected radiation, which depends on the different refractive indexes of the IRE crystal and the sample at different frequencies of the interacting light. Shifts in band positions are thus optical effects caused by changes in the refractive index.^64^Figure 1 also contains results of the curve fitting procedure of the SEIRA spectra in the spectral range 1650–1250 cm^–1^. This method is advantageous for highlighting small relative shifts in the wavenumbers of bands and allows the separation of overlapping bands.

**Table 1:** Assignment of the SEIRA Bands in the Spectral Region below 1250 cm^–1^ a

S-containing amino acids		His		Phe/Tyr/Trp	
assignment	cm^–1^	assignment	cm^–1^	assignment	cm^–1^
ν(C–C), ρ_ipb_(CSH)	987	ρ_ω_(NH), δ(ring), ν(C–N)	956	ν(C–COO^–^)	939
ρ_ipb_(CNH)	943	δ(ring), ν(C–N)	908	ν(C–C)	895
ν(C–C)	872	ρ_ω_(CH), ρ_τ_(ring)	823	δ_oop_(CH)_ring_	877
ν(C–C), ν(C–S), ρ_ipb_(CSH)	823	ν(C–C/N), δ(ring),	667	Fermi doublet	840/827
ρ_ω_(COO^–^)	661	ν(C–C/N), δ(ring)	625	ν skeletal	792
δ_oop_(COO^–^)	533	ρ_r_(COO^–^)	524	δ_oop_(CH)_ring_	739
δ_oop_(C=O)	513			δ_oop_(CH)_ring_	713
				δ_ip_(CH)_ring_	574
				δ_oop_(COO^–^)	527

a Abbreviations: ν, stretching; ρ_ipb_, in-plane bending; ρ_ω_, wagging; ρ_r_, rocking; ρ_τ_, twisting; δ, deformation; δ_oop_, out-of-plane deformation; δ_ip_, in-plane deformation vibrations.

Since dipole moments of polar bonds such as O−H, C=O, and N−H (found in amide bonds and functional groups) change the most during the vibrations of the molecule, they produce strong bands in the infrared spectra, most of which can be observed in the SEIRA spectrum of Cys on AuNPs (Figure 1A, green line trace) at similar wavenumbers as in the corresponding ATR-FTIR spectrum (Figure 1A, black line trace). In contrast, the thiol group (−CSH) adsorbs radiation in the IR range very poorly (low dipole moment of the C–S and S–H bonds), and thus the vibrations of this group yield weak bands, of which only the ν(S–H) mode can be unambiguously assigned despite its low intensity, due to its occurrence in the unambiguous spectral range (2400–2600 cm^–1^).

For Cys deposited on the AuNPs surface (Figure 1A, green line trace), the largest wavenumber shift is observed at 661 cm^–1^ [ν(C–S) of P_H_-T conformer] (ATR-FTIR, at 693 cm^–1^, P_C_-G conformer; where P_C_ and P_H_ refer to the two possible conformations of the CH_2_–CH_2_–S moiety with the C and H atoms in *trans* position to the sulfur atom, respectively, whereas T and G stand for *trans* and *gauche* internal rotation around the CH_2_–S bond) and 1511^c^ cm^–1^ [(ρ_symb_(NH_3_^+^)] (ATR-FTIR, at 1525^c^ cm^–1^) (where ^c^ denotes curve-fitted bands). The curve fit also indicates that 1511^c^, 1482^c^ [(ρ_symb_(NH_3_^+^)], 1380^c^ [ν_sym_(COO^–^)], 1360^c^ [δ(CH)], and 1326^c^ cm^–1^ [(ρ_w_(CH_2_)] SEIRA signals increase in intensity compared to the corresponding ATR-FTIR bands. In contrast, 1423 [ρ_b_(CH_2_)], 1064 [ρ_ipb_(SH)], 870 [ρ_w_(COO^–^)], 661, 634 [ρ_w_(COO^–^)], 533 [δ_oop_(COO^–^)/ρ_b_(CH–SH)], and 448 cm^–1^ [ρ_b_(CH_2_–CH–SH)] spectral features decrease in intensity. Two more bands at 987 [ν(CC)/ρ_s_(NCH)] and 513 cm^–1^ [ρ_b_(CH_2_–CH–N)] appear in the SEIRA spectrum of Cys on AuNPs. On the basis of the above information, it can be concluded that Cys is adsorbed on the surface of AuNPs: (1) mainly via the −NH_3_^+^ group, (2) the −COO^–^ and −SH groups are involved in the interaction of Cys with AuNPs, and (3) as a result of adsorption, a conformational change of the thiol group occurs. The presence and intensity (higher than that in the ATR-FTIR spectrum) of the 2541 cm^–1^ band indicate that the thiol group on AuNPs is not deprotonated, and the free electron pair on sulfur has contact with the AuNPs surface, which means that the C–S bond (knowing that sulfur has sp^3^ hybridization) is tilted toward the AuNPs surface.

More spectral differences can be seen between the ATR-FTIR spectrum (Figure 1A, black trace) and the SEIRA spectrum of Cys on AgNPs (Figure 1A, blue line trace). These differences relate to changes in both the intensity and wavenumber of the observed bands. For example, spectral features at 1558^c^ [ρ_symb_(NH_3_^+^)], 1511^c^, 1404^c^ [ρ_s_(CH_2_)], 1381^c^, 1336^c^ [ρ_symb_(NH_3_^+^)], and 1295^c^ cm^–1^ [ρ_w_(CH_2_)] lose their SEIRA intensity most significantly. The ATR-FTIR bands at 2549, 823 [ν(C–C/S)/ρ_s_(CSH)], 753 [ν(C–S)_*Pc*-G_], and 636 cm^–1^ [ν(C–S)_PH-T_] disappear. A new weak SEIRA band at 613 cm^–1^ [ν(CH–COO^–^)] appears. Some other bands shift in wavenumber roughly maintaining their intensity, e.g., 1141_ATR-FTIR_ → 1125_SEIRA_ cm^–1^ [ρ_r_(NH_3_^+^)], 940_ATR-FTIR_ → 962_SEIRA_ cm^–1^ [ν(N–C)], 865_ATR-FTIR_ → 845_SEIRA_ cm^–1^, 692_ATR-FTIR_ [ν(C–S)_Pc-G_] → 674_SEIRA_ cm^–1^ [ν(C–S)_PH-T_]. Given this, it appears that the thiol group of Cys, which adopts one conformation (the same as on the AuNPs surface), is deprotonated on AgNPs and the contact between the amine/carboxylate groups and the surface of AgNPs is weakened.

Two other sulfur-containing amino acids differ from Cys in that cystine is an l-cysteine dimer formed by a disulfide bond, and the side chain of Met is two carbon atoms longer (by −CH_2_– and terminal −CH_3_ units) than the side chain of Cys. Therefore, bands with similar vibrations are expected in the SEIRA spectra of cystine and Met. In general, the SEIRA spectra of cystine on AuNPs (Figure 1B, green line trace) and AgNPs (Figure 1B, blue trace) contain the same set of bands as the corresponding ATR-FTIR spectrum (Figure 1B, black trace), suggesting that the terminal groups and side chain of this peptide interact with the surface of both metals. However, for cystine on AuNPs, the spectral features at 1619^c^ [(ρ_asymb_(NH_3_^+^)] and 1570^c^ cm^–1^ and in the spectral region below 1350 cm^–1^ show higher and similar SEIRA intensities as the corresponding ATR-FTIR intensities except for the weak bands at 1245 [ρ_t_(CH_2_)], 649 [ν(C–S)_PH-T_], and 573 cm^–1^ [ρ_t_(NH_3_^+^)], which appear only in the SEIRA spectrum of cystine adsorbed on the surface of AuNPs. In the above spectral region, for cystine on AgNPs, the SEIRA signals are weaker than the corresponding bands for this amino acid deposited on AuNPs except for the spectral feature at 1090 cm^–1^ [ρ_r_(NH_3_^+^)], which has a comparable intensity in all cystine spectra shown. Bands at 1616^c^ and 1577^c^ cm^–1^ are the strongest for cystine on AgNPs, while bands at 1482^c^ and 1453^c^ cm^–1^ are the weakest for cystine on AgNPs. All these changes indicate the following: (1) the presence of cystine in one rotamer on AgNPs (at 757 cm^–1^, P_C_-G) and two C–S rotamers on AuNPs (at 757 and 649 cm^–1^) while maintaining the same C–S–S–C fragment conformation (dihedral angle) as in aqueous solution, as evidenced by the 538 cm^–1^ band of the *trans–gauche–trans* (T*G*′T) conformer, (2) a strong interaction between cystine and the surface of AuNPs, and (3) a weak interaction of cystine with AgNPs. A slightly larger shift in the wavenumbers for cystine on AuNPs (e.g., 1617 and 1084 cm^–1^ bands by −4 cm^–1^) compared to cystine on AgNPs (e.g., 1620 and 1087 cm^–1^ bands by −1 cm^–1^) confirms the differences in the strength of the interaction between cystine and metal surfaces used.

One of the distinguishing features of ATR-FTIR (Figure 1C, black line trace) of SEIRA spectra of Met adsorbed on the surface of AuNPs (Figure 1C, green trace) and AgNPs (Figure 1C, blue trace) is the full width at half-maximum (fwhm) of the 1507 cm^–1^ band. The fwhm of this band decreases dramatically in the SEIRA spectra (fwhm_ATR-FTIR_ = 49 cm^–1^, fwhm_AuNPs_ = 15 cm^–1^, and fwhm_AgNPs_ = 20 cm^–1^), and the band shape becomes symmetric compared to the asymmetric shape of the corresponding ATR-FTIR band. The second derivative of the Met ATR-FTIR spectrum shows three components hidden under this band: i.e., one intense at 1511 cm^–1^ and two shoulders at 1497 and 1487 cm^–1^. According to work by Cao and Fisher, these bands can be assigned to the ρ_symb_(NH_3_^+^) modes of different conformers of the −H_2_C–S– unit.^62^ It can be concluded that the side chain of Met in the solid-state adopts multiple conformations of the −H_2_C–S– unit, whereas it exists as only one rotamer for the Met adsorbed on both metal surfaces.

Other significant spectral differences relate to the intensity of the bands. For example, the intensity of the overlapping 1606, 1582, and 1560 cm^–1^ ATR-FTIR spectral features (Figure 1C, black line trace) varies from the lowest to the highest in the direction of the lower wavenumbers, and the band at 1510 cm^–1^ has a similar strength to the 1582 cm^–1^ band. In the SEIRA spectra of Met, the envelope of the overlapping bands does not change significantly in intensity, maintaining the following relative intensities the ∼1580^c^ cm^–1^ SEIRA spectral feature is (1) more intense than the other two bands from the envelope of the overlapping bands (slightly gaining in strength for Met on AuNPs (Figure 1C, green line trace)) and (2) less intense than the ∼1507^c^ cm^–1^ SEIRA signal, which is the strongest band in the spectrum of Met on AgNPs (Figure 1C, blue line trace). In the spectral region below 1250 cm^–1^, all Met SEIRA signals are weaker than the corresponding ATR-FTIR bands. Of these bands, the spectral features at 1241, 1184 [ρ_b_(CH_2_)], 1150, 1118 [ν(C–N)/ρ_s_(CNH)], 980, 874, and 542 cm^–1^ are clearly visible. All these bands are due to the vibrations of the N-terminal group, and therefore this fragment is responsible for the direct interaction of Met with the surface of the two metallic NPs, which is slightly stronger for Met on AgNPs than for Met on AuNPs. However, the weak 1632 and 539 cm^–1^ bands might indicate that the carboxyl group is located at some distance from the metallic surfaces.

### Aromatic Amino Acids

**Figure 2:**
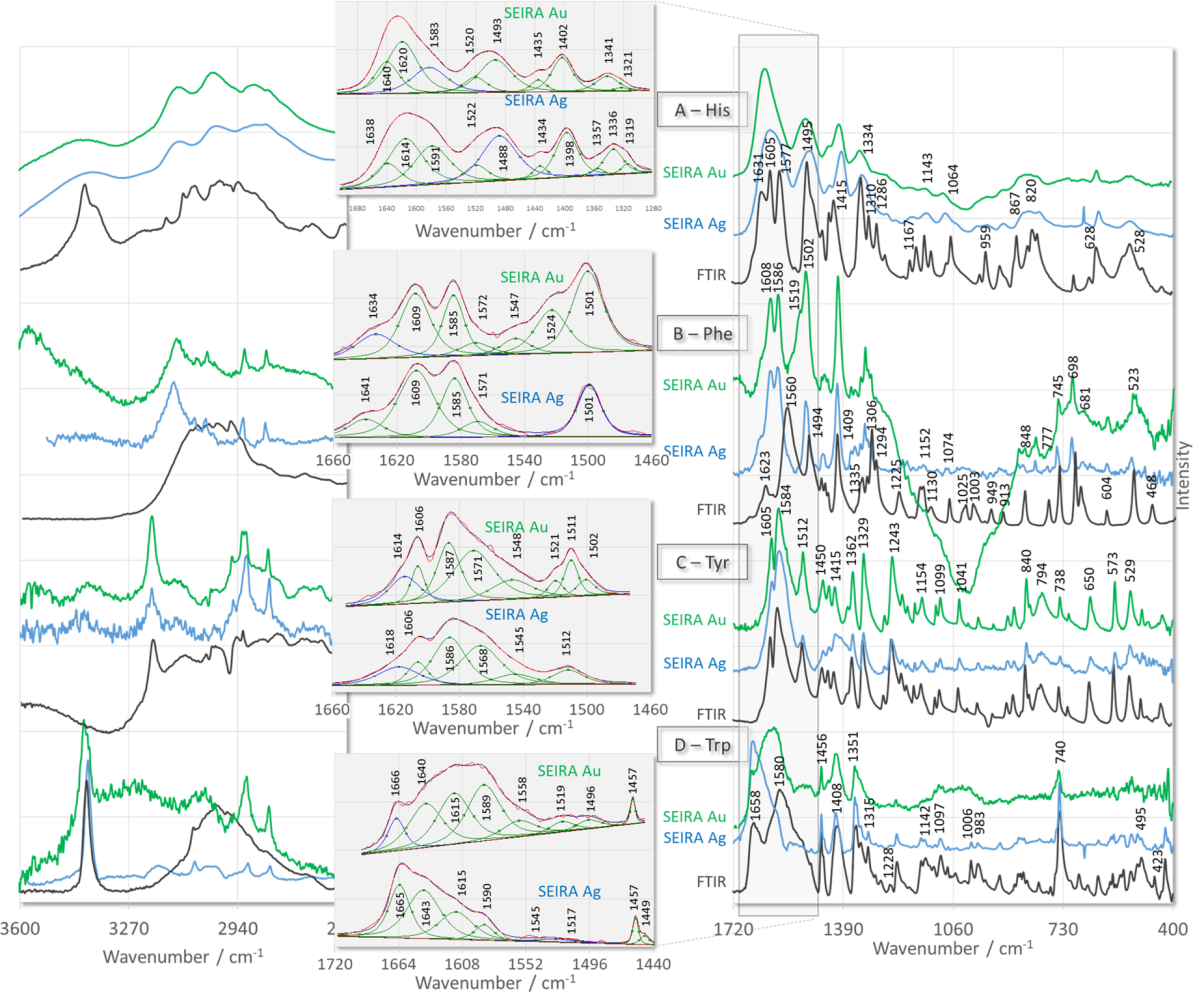
ATR-FTIR (black line traces) and SEIRA (with curve fitting results) spectra of aromatic amino acids adsorbed onto the surface of AgNPs (blue line traces) and AuNPs (green line traces).

l-Histidine (His) SEIRA spectra (Figure 2A, green and blue line traces) show differences from the ATR-FTIR spectrum (Figure 2A, black line trace). To explain this phenomenon, one must consider the nature of His (its five ionic forms, each of which has characteristic bands due to the vibrations of the functional groups and the imidazole ring) and the surface selection rule for metal surfaces, which states that only the modes with nonzero dipole moment derivative components perpendicular to the surface are infrared active.^3^ This rule was formulated based on the observation of the induced image dipoles. In short, a change in the dipole moment parallel to the surface is canceled by an equal change in the opposite direction of the dipole moment induced in the substrate, while the change in the dipole moment perpendicular to the surface is enhanced and the total dipole moment is doubled.

In the SEIRA spectra of His on AuNPs (Figure 2A, green line trace) and AgNPs (Figure 2A, blue line trace), the 1640^c^ [ρ_asymb_(NH_3_^+^)], 1620^c^ [ν_asym_(COO^–^)], 1583^c^ [ν(ring) + ρ_ipb_(N_1_H)], 1520^c^ [ν_asym_(C–N_1,3_/C)], 1493^c^ [ν(ring) + ρ_ipb_(N_1_H)], 1435^c^ [δ(CH_2_) + δ(N_1_H)], 1402^c^ [ν_sym_(COO^–^)], 1341^c^ [δ(N_1_H)], and 1321^c^ cm^–1^ [ρ_t_(N_1_H)] bands are mainly enhanced. The wavenumbers of these SEIRA signals indicate the zwitterionic form of His with a neutral imidazole ring (N_1_-protonated).^60,65,66^ Of these bands, the spectral features at 1591 and 1493 cm^–1^ are more intense on AgNPs compared to AuNPs, and the bands at 1357 [ν(C−N)_ring_] and 668 cm^–1^ [ρ_oopb_(ring)] appear only for His adsorbed on AgNPs. It can be concluded that the same molecular fragments of His interact with the surface of both substrates, but either the strength of Ag/Au···N_1_–H interactions or the arrangement of the imidazole ring to the substrate surface is slightly different on the two surfaces.

The ATR-FTIR spectrum of l-phenylalanine (Phe) shown in Figure 2B (black line trace) is consistent with the FTIR spectrum published by Wolpert and Hellwing.^60^ Spectral differences between this spectrum and the SEIRA spectra of Phe adsorbed on AuNPs (Figure 2B, green line trace) and AgNPs (Figure 2B, blue line trace) indicate a specific interaction of Phe molecular fragments with the surface of both substrates. The fragments interacting with the surface of AgNPs and AuNPs are the phenyl ring and the carboxylate group, as shown by the prominent bands at 1609^c^ [ν_8a_], 1585^c^ [ν_8b_], 1501^c^ [ν_19a_], 748 [ν_11_], and 703 cm^–1^ [ν_4_] and at 1634^c^ [ν_asym_(COO^–^)], 1408^c^ [ν_sym_(COO^–^)], 1325^c^ [ρ_oopb_(CH_2_)], and 523 cm^–1^ (ρ_r_(COO^–^) + δ(C=O)], respectively. In the spectrum of Phe on AuNPs, the 1547^c^ [ρ_asymb_(NH_3_^+^)] and 1524^c^ cm^–1^ [ρ_symb_(NH_3_^+^)] SEIRA signals are also observed, indicating a protonated amino group that helps in the adsorption of Phe on the AuNPs surface.

Considering the various possibilities of Tyr adsorption on metallic surfaces,^67,68^ the wavenumbers of the ν_8a_ (at 1606^c^ cm^–1^) and ν_8b_ (at about 1570^c^ cm^–1^) modes in the Tyr spectra shown in Figure 2C (green and blue line traces) indicate the presence of Tyr in the tyrosinate (TyrO^–^) form. The 1245 cm^–1^ spectral feature attributed to the ν(C_Ph_–O) [ν_7a_] vibrations confirms the previous statement, although TyrO^–^ does not undergo chemisorption on the surface of AgNPs and AuNPs, either via the π-electron system or via the free-electron pair at the phenolic oxygen. This is because no significant shift in wavenumbers (ATR-FTIR vs SEIRA) is observed (Δν = −2 cm^–1^) and the AgNPs/AuNPs···TyrO^–^ interaction (via the phenolic oxygen with the vertical arrangement of the phenolic ring) is a sufficient reason for the broadening of the SEIRA bands (ΔFWFM = 7–9 cm^–1^). The amino and carboxylate groups of Tyr also interact with AuNPs (Figure 2C, green line trace) and AgNPs (Figure 2c, blue line trace), as indicated by strong bands at approximately 1586^c^, 1548^c^, 1511^c^, and 1154 cm^–1^ [ρ_r_(NH_3_^+^)] and at approximately 1734^c^ [ν(C=O)], 1415^c^ [ν_sym_(COO^–^)], 649 [ρ_ω_(COO^–^)], 573 [ρ_ipb_(NH_3_^+^) + δ(COO^–^)], and 529 cm^–1^, respectively. However, there are differences in the intensity of the bands due to the amine and carboxylate groups and phenyl ring vibrations for TyrO^–^ on AgNPs compared to those for TyrO^–^ on AuNPs. Briefly, 1243, 1329, 1362, and 1415 cm^–1^ bands in the spectrum of TyrO^–^ on AgNPs are 3 times less intense than the 1587 cm^–1^ SEIRA signal, and the bands in the wavenumber region below 900 cm^–1^ show even lower intensity, while the 1586 cm^–1^ band in the spectrum of TyrO^–^ on AuNPs is less than 2 times more intense than the bands assigned to the −COO^–^ and phenyl ring vibrations. These fluctuations indicate that Tyr interacts directly with the AgNPs surface via the −NH_3_^+^ group. Moreover, M. Osawa et al. have shown that when the – COO^–^ group is bound to the metal surface as a bidentate coordination ligand, the ν_s_(COO^–^) mode is enhanced, while the ν_as_(COO^–^) mode is not enhanced compared to the same bands in the IR spectrum.^69^ From these results, it can be concluded that the bidentate coordination of the *C*-terminal group of TyrO^–^ is present on both metal surfaces.

The comparison of the spectral region below 1500 cm^–1^ in the ATR-FTIR spectrum of l-tryptophan (Trp) (Figure 2D, black line trace) with the SEIRA spectrum of Trp adsorbed on the AgNPs surface (Figure 2D, blue line trace) suggests that the same set of bands is observed in these spectra, and the SEIRA signals have much lower intensity than the ATR-FTIR bands except for the spectral features at 1457^c^ [ρ_symb_(NH_3_^+^)], 1449^c^ [W6, ν_sym_(N_1_C_2_C_3_)], 1414^c^, 1356 [W7, ν(N_1_H) + δ_oop_(ring)], and 743 cm^–1^ [W18, indole ring breathing].^60,69,70^ In the wavenumber range of 1700–1500 cm^–1^, the intensities of the 1665^c^ [ρ_asymb_(NH_3_^+^)] and 1643^c^ cm^–1^ [ν_asym_(COO^–^)] bands increase significantly in terms of the ATR-FTIR intensity, while the 1590^c^ [W2], 1545^c^ [W3, ν(C_2_=C_3_)], and 1517^c^ cm^–1^ [ν(pyrrole)] SEIRA signals decrease significantly in intensity. On the basis of the above observations, it can be assumed that Trp adsorbs on AgNPs via the terminal groups and pyrrole nitrogen of the indole ring. On the other hand, in the SEIRA spectrum of Trp on AuNPs (Figure 2D, green trace), the 1640^c^ and 1615^c^ cm^–1^ [ν(phenyl)] bands increase in intensity, the 1558^c^, 1519^c^, 1351, and 740 cm^–1^ bands decrease in intensity, and 1449^c^ and 1316 cm^–1^ [ν(pyrrole)] SEIRA signals disappear compared to the ATR-FTIR spectrum. Thus, Trp interacts with the surface of AuNPs via the −COO^–^ and −NH_3_^+^ groups and the phenyl ring of indole.

### Neurotransmitters

There are few literature reports on the IR studies of the tested peptides,^71−73^ and the results of the SEIRA studies are not available. Structural information for these peptides can be obtained by analyzing the amide bands, particularly amide I, II (of relatively strong infrared intensity), and III (Raman-active), whose wavenumbers are sensitive to peptide chain conformation (e.g., α-helices, β-sheets, turns, and disordered structure) and hydrogen bonding in the peptide backbone.

**Figure 3:**
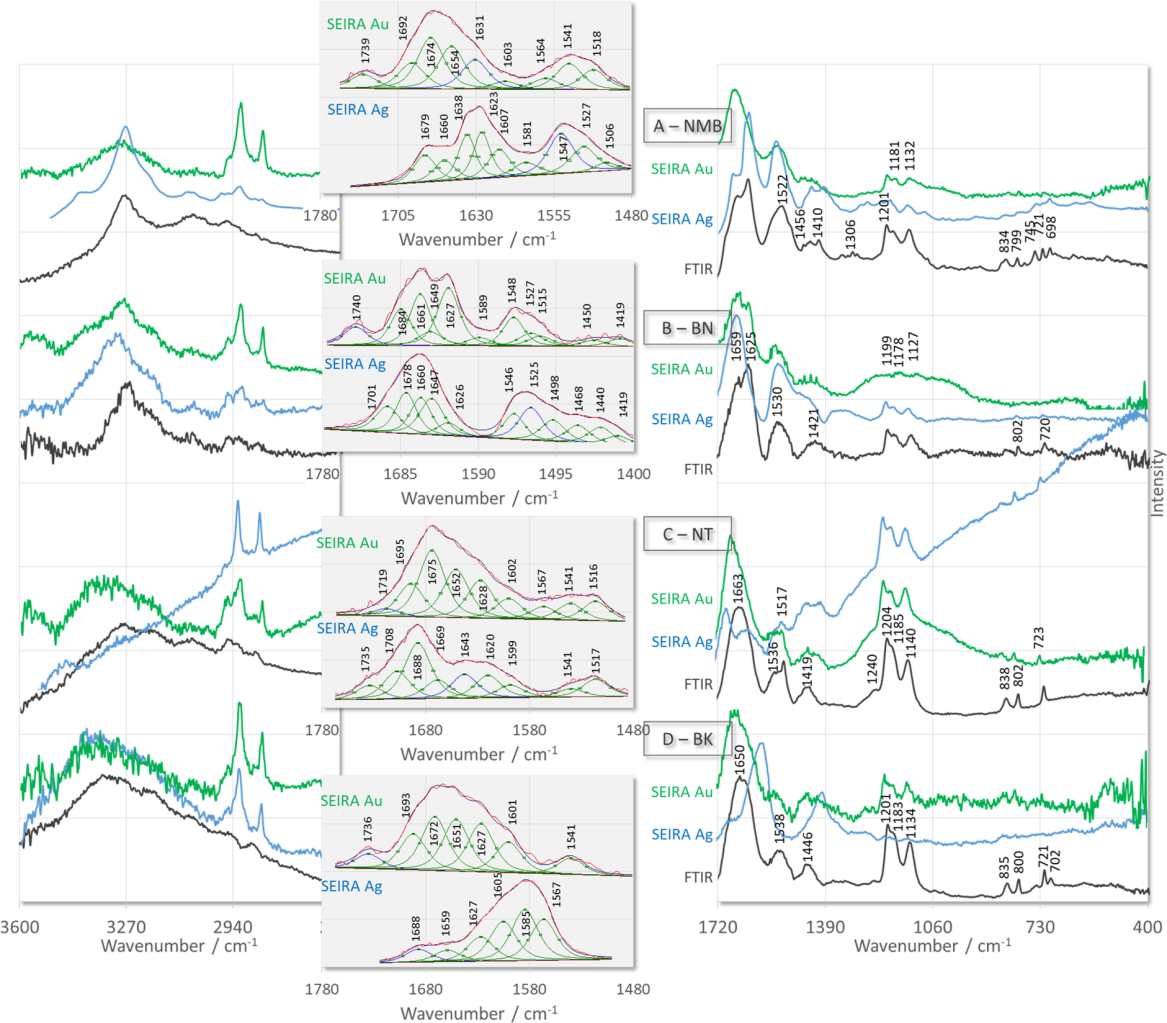
ATR-FTIR (black line traces) and SEIRA (with curve fitting results) spectra of the investigated neurotransmitters adsorbed onto the surface of AgNPs (blue line traces) and AuNPs (green line traces).

As shown in Figure 3, the width of the contributing component bands within the amide I and II regions (above 1500 cm^–1^) is greater than the distance between the maxima of adjacent bands. As a consequence, the individual component bands cannot be separated in the experimental spectra. The curve-fitting procedure for these regions allowed us to increase the separation of the overlapping components present in the broadband envelope. For the SEIRA spectra of NMB on AuNPs (Figure 3A, green line trace) and AgNPs (Figure 3A, blue line trace), the fitting results show multiple components at 1739^c^, 1692^c^, 1674^c^, 1654^c^, 1631^c^, 1603^c^, 1564^c^, 1541^c^, and 1518^c^ cm^–1^ and at 1679^c^, 1660^c^, 1638^c^, 1623^c^, 1607^c^, 1581^c^, 1547^c^, 1527, and 1506 cm^–1^, respectively. Considering the primary structure of this peptide (Table 2), these bands are due to the vibrations of the amide bonds (amide I and II), the −CONH_2_ unit of Asn, and the phenyl and indole rings of Phe and Trp. Bands of His are also expected in this wavenumber range, e.g., at 1583^c^ cm^–1^, but this band is not present in the SEIRA spectrum of NMB on AuNPs, suggesting that His is not localized near this surface. In the case of NMB adsorption on the AgNPs surface, the SERS signal is observed at 1581 cm^–1^. Unfortunately, this band cannot be unambiguously assigned to imidazole vibrations, since it occurs at the wavenumber characteristic of phenyl (co)ring (Phe/Trp) vibrations. Considering that in the spectrum of NMB on AgNPs it is difficult to identify other bands attributed to imidazole vibrations, while the phenyl (co)ring vibrations give spectral feature at 1607^c^, 1547^c^, and 1506^c^ cm^–1^, the intensity and width of the band at 1581 cm^–1^ suggest that it is associated with the phenyl ring vibrations rather than the imidazole ring vibrations.

**Table 2:** Amino Acid Sequence of the Investigated Neurotransmitters^a^

	amino acid sequence														
	1	2	3	4	5	6	7	8	9	10	11	12	13	14	
NMB	Gly	Asn	Leu	**Trp**	Ala	Thr	Gly	**His**	**Phe**	Met	NH_2_				
BN	pGlu	Gln	Arg	Leu	Gly	Asn	Gln	**Trp**	Ala	Val	Gly	**His**	Leu	Met	NH_2_
NT	pGlu	Leu	**Tyr**	Glu	Asn	Lys	Pro	Arg	Arg	Pro	**Tyr**	Ile	Leu	OH	
BK	Arg	Pro	Pro	Gly	**Phe**	Ser	Pro	**Phe**	Arg						

a pGlu represents 5-oxoproline.

**Figure 4:**
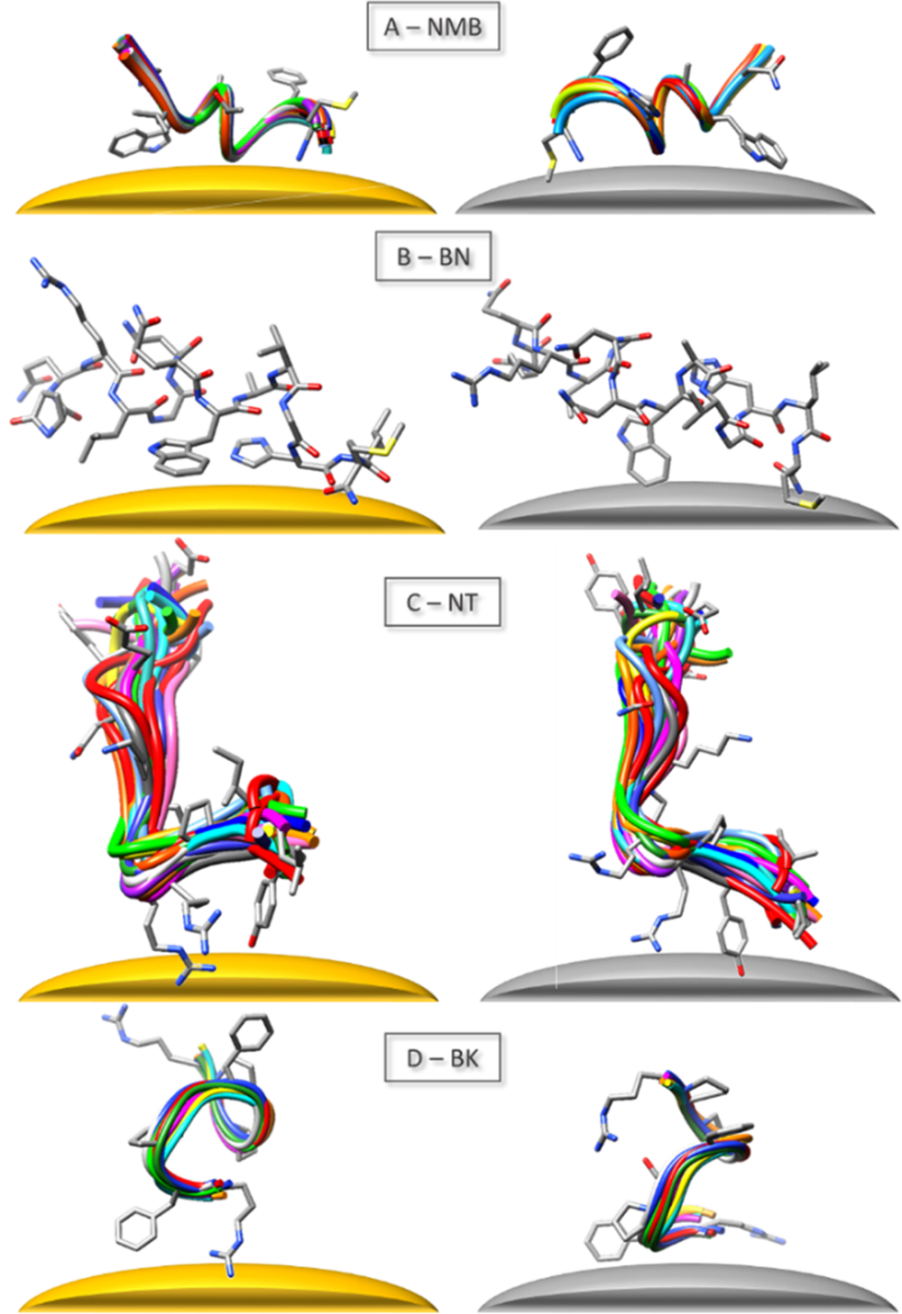
Proposed manner of adsorption on the surface of AuNPs (on left) and AgNPs (on right) for the investigated peptides.

The SEIRA signals at 1739^c^ cm^–1^ for NMB on AuNPs and at 692 and 745 cm^–1^ for NMB on AgNPs suggest that the C=O group of Asn and the −S–CH_3_ fragment of Met interact with AuNPs and AgNPs, respectively. Thus, using the determined NMB structure (see Figure 4), it can be assumed that the 1607^c^, 1581^c^, 1547^c^, and 1506^c^ cm^–1^ SEIRA signals for NMB on AgNPs are caused by the indole ring vibrations, and the 1679 and 1623 cm^–1^ bands are due to the vibrations of the amidated *C*-termini. This implies that these fragments are involved in the adsorption of NMB on AgNPs (similar conclusions were drawn based on the results of SERS^74^), and the assignment of the remaining eight bands is as follows: δ(NH_2_), amide I (random structure), amide I (α structure), δ(NH_2_), W1, amide II, and W3. This band assignment again suggests that Asn and Trp are in contact with AuNPs. Figure 4A shows the proposed mode of interaction of NMB with the surfaces of AgNPs and AuNPs.

1740^c^, 1684^c^, 1661^c^, 1649^c^, 1627^c^, 1589, 1548^c^, 1527^c^, and 1515^c^ cm^–1^ and the 1701^c^, 1678^c^, 1660^c^, 1647^c^, 1626^c^, 1546^c^, 1525^c^, and 1498^c^ cm^–1^ SEIRA signals in the spectra of BN, deposited on AuNPs (Figure 3B, green line trace) and AgNPs (Figure 3B, blue line trace), respectively, can be assigned analogously to those observed in the NMB SEIRA spectra. The absence of ν(C–S) in the SEIRA spectrum of BN on AuNPs and its very weak intensity for BN on AgNPs (at 721 cm^–1^) also suggest that Met is close to the surface of AgNPs and the Met and His side chains are on the opposite side of the peptide chain (a crystallographic structure is not available). On the basis of the above observations and considering the proposed BN structure (Figure 4B), one can propose a type of BN arrangement on the substrate surfaces.

For NT on AuNPs (Figure 3C, green line trace) and AgNPs (Figure 3C, blue line trace), 1719^c^, 1695^c^, 1667^c^, 1652^c^, 1628^c^, 1602^c^, 1567^c^, 1541^c^, and 1516^c^ cm^–1^ and the 1735^c^, 1708^c^, 1688^c^, 1669^c^, 1643^c^, 1620^c^, 1599^c^, 1541^c^, and 1517^c^ cm^–1^ SEIRA signals were fitted, respectively. These bands are due to the vibrations of the amide bond, C=O moiety, and amino/guanidine groups. In the case of this peptide, the Arg residues are located at positions 8 and 9 of the amino acid sequence of NT. Therefore, it can be assumed that Tyr at position 11 interacts with the surface (Figure 4). The wavenumbers of the ν_8a_ and ν_8b_ modes of Try indicate that TyrO^–^ is in contact with the surface of AuNPs (at 1602^c^ and 1567^c^ cm^–1^), whereas TyrOH is close to the AgNPs surface (at 1620^c^ and 1599^c^ cm^–1^).

For BK, adsorbed on the surface of AuNPs (Figure 3D, green line trace), 1736^c^, 1693^c^, 1672^c^, 1651^c^, 1627^c^, 1601^c^, and 1541^c^ cm^–1^ bands were fitted. Undoubtedly, the 1736^c^ cm^–1^ SEIRA signal is due to ν(C=O) of the *C*-terminus. Assuming an interaction between the C=O group and the AuNPs surface, one can expect bands assigned to the peptide bonds (at 1651^c^ and 1541^c^ cm^–1^) and to the vibrations of the guanidine moiety (at 1693^c^, 1672^c^, and 1627^c^ cm^–1^), which is the case. The last of the calculated bands (at 1601^c^ cm^–1^) indicates the presence of the phenyl ring in contact with AuNPs. Similarly, for BK on AgNPs (Figure 3D, blue line trace), the 1605 and 1585 cm^–1^ indicate the involvement of the phenyl ring in the peptide interaction with AgNPs. The absence of ν(C=O) indicates that BK adopts an orientation on the surface of AgNPs in which only the guanidine and the phenyl ring interact with AgNPs (Figure 4D). The intensity of the fitted Tyr bands is stronger on AgNPs than on AuNPs for BK, while the vibrations of the guanidine group are more intense on AuNPs. This indicates that on AgNPs mainly the Phe ring is involved in the peptide interaction with the substrate surface, while on AuNPs mainly the Arg side chain of BK adsorbs.

## Conclusions

NMB, BN, NT, and BK are important neurotransmitters found in body fluids and are known as tumor growth factors. When bound to metal nanoparticles, they have potential applications in tumor imaging and anticancer therapy. Therefore, it is important to understand their adsorption on the surface of metal NPs. Information on adsorption can be obtained using the SERS and SEIRA methods, which complement each other and allow a comprehensive analysis.

In this work, we present SEIRA results for the above neurotransmitters immobilized on the surface of readily available and homogeneous, in terms of shape and diameter, Ag and Au nanoparticles, which may bring us closer to the more frequent and routine application of SEIRA. Analysis of SEIRA spectra of peptides was possible because of curve fitting of these spectra and SEIRA data for selected amino acids (e.g., sulfur-containing and aromatic). On this basis, it was shown that peptides adsorb differently on the two metal surfaces via molecular fragments located on the *C*-terminal part of the chain. Briefly, in the case of NMB, the peptide bond(s) and the Trp ring are in contact with the surfaces of both metals, but the amidated *C*-terminus or side chain of Met interacts with AuNPs and AgNPs, respectively. In the case of BN, the indole and imidazole rings, the amidated *C*-terminus, and the peptide bond(s) are responsible for the interaction with AgNPs, while the side chains of Trp and Met and the peptide bond(s) are on or near AuNPs. The side chains of Tyr and Arg interact with both metallic surfaces but in a different state of protonation of the hydroxyl group (TryO^–^ on AuNPs and TyrOH on AgNPs). Phe, Arg, and the C-terminus are responsible for the adsorption of the last peptide studied (BK) on the surface of AuNPs, but Arg and Phe are in contact with AgNPs.

The SEIRA results also showed that the investigated peptides change their structure upon adsorption and interact with the surfaces of AuNPs and AgNPs with amino acids residues located in the *C*-terminal part of the peptide chain; similar conclusions were drawn from the studies of the biological activity of these peptides.^75−87^ Thus, evidence has been obtained confirming the validity of SEIRA to select those peptide fragments within the study group of peptides that play a role in the substrate–receptor interaction in systems where biological studies are difficult or do not lead to the unambiguous determination of the peptide fragments responsible for its biological activity.

## Supporting Information Available

Detailed description of the procedure for obtaining the investigated peptides (PDF)

## Abbreviations

NMB: neuromedin B
BN: bombesin
NT: neurotensin
BK: bradykinin
ATR-FTIR: attenuated total reflection Fourier transform infrared spectroscopy
SEIRA: surface-enhanced infrared spectroscopy
