# Supplementary material for: Is the Use of Surface-Enhanced Infrared Spectroscopy Justified in the Selection of Peptide Fragments That Play a Role in Substrate–Receptor Interactions? Adsorption of Amino Acids and Neurotransmitters on Colloidal Ag and Au Nanoparticles
Source: J Phys Chem B. 2021 Mar 1;125(9):2328–38. doi: 10.1021/acs.jpcb.1c00546 (PMC8041316; doi:10.1021/acs.jpcb.1c00546)
Supplement: Supplementary file 1 — jp1c00546_si_001.pdf [file jp1c00546_si_001.pdf]

Supporting Information

Is the use of surface-enhanced infrared spectroscopy justified in the selection of peptide fragments that play a role in substrate-receptor interactions? Adsorption of amino acids and neurotransmitters on colloidal Ag and Au nanoparticles.

E. Proniewicz[a], A. Tąta[a], E. Iłowska[b], A. Prahl[b]*

[a] Faculty of Foundry Engineering, AGH University of Science and Technology, 30-059 Krakow, Poland

[b] Faculty of Chemistry, University of Gdansk, Wita Stwosza 63, 80-308, Gdansk, Poland

MATERIALS AND METHODS

Adsorbates and Colloids.

Neuromedin B (NMB), bombesin (BN), neurotensin (NT), and bradykinin (BK) were synthesized via the solid-phase method using the Fmoc strategy and starting from Fmoc-Wang resin (GL Biochem Shanghai, 1% DVB, 100–200 mesh). The load of the resin for the first protected amino acid (Fmoc-protected amino acids) was 0.40 mmol/g. Fmoc protection group was removed by 20% piperidine in DMF. A threefold excess of the respective Fmoc-amino acids was activated in situ using HATU (1 eq)/HOAt (1 eq) in a mixture of DMF/NMP (1:1 v/v) that contained 1% Triton, and the coupling reactions were base-catalyzed with NMM. All of the Fmoc-protected amino acids were purchased from commercial sources (NovaBiochem, Bad Soden, Germany). Cleavage of the peptide from the resin with side-chain deprotection was performed by treatment with trifluoroacetic acid (TFA):H_2O:TIS (95.5:2.5:2.5 v/v/v) for 4 h. The total volume of the TFA filtrate was reduced to approximately 1 ml by evaporation in vacuo. The peptide was precipitated with cold diethyl ether and filtered through a Schott funnel. Peptides were purified by semi-preparative high-performance liquid chromatography (HPLC). HPLC was performed on a Waters (analytical and semi-preparative) chromatograph equipped with a UV detector (λ = 226 nm). The purity of the peptides was determined on a Discovery HS C_{18} column (5μm, 100 Å; 250 × 4.6 mm). The solvent systems were [A] 0.1% aqueous TFA and [B] 80% acetonitrile in aqueous 0.1% TFA (v/v). A linear gradient from 1 to 80% of [B] for 30 min was used for peptides at a flow rate of 1 ml/min. semi-preparative HPLC was performed using a Kromasil C_8 column (5μm, 100 Å; 16 × 250 mm) in a linear gradient of [B] from 13 to 43% at a flow rate of 8 ml/min. The mass spectra of peptides were recorded on a

Bruker BIFLEX III MALDI TOF mass spectrometer with ionization by a 337 nm nitrogen laser line.
